# A Systematic Review on Common and Distinct Neural Correlates of Risk-taking in Substance-related and Non-substance Related Addictions

**DOI:** 10.1007/s11065-022-09552-5

**Published:** 2022-07-30

**Authors:** Philippa Hüpen, Ute Habel, Mikhail Votinov, Joseph W. Kable, Lisa Wagels

**Affiliations:** 1 Department of Psychiatry, Psychotherapy and Psychosomatics, Faculty of Medicine, RWTH Aachen, Aachen, Germany; 2 JARA - Translational Brain Medicine, Aachen, Germany; 3 Institute of Neuroscience and Medicine: JARA-Institute Brain Structure Function Relationship (INM 10), Research Center Jülich, Jülich, Germany; 4 Department of Psychology, University of Pennsylvania, Philadelphia, PA 19104 USA

**Keywords:** Decision-making, Risk-taking, Uncertainty, Addiction, Behavioral addiction, Neural correlates

## Abstract

Both substance-related as well as non-substance-related addictions may include recurrent engagement in risky actions despite adverse outcomes. We here apply a unified approach and review task-based neuroimaging studies on substance-related (SRAs) and non-substance related addictions (NSRAs) to examine commonalities and differences in neural correlates of risk-taking in these two addiction types. To this end, we conducted a systematic review adhering to the PRISMA guidelines. Two databases were searched with predefined search terms to identify neuroimaging studies on risk-taking tasks in individuals with addiction disorders. In total, 19 studies on SRAs (comprising a total of 648 individuals with SRAs) and 10 studies on NSRAs (comprising a total of 187 individuals with NSRAs) were included. Risk-related brain activation in SRAs and NSRAs was summarized individually and subsequently compared to each other. Results suggest convergent altered risk-related neural processes, including hyperactivity in the OFC and the striatum. As characteristic for both addiction types, these brain regions may represent an underlying mechanism of suboptimal decision-making. In contrast, decreased DLPFC activity may be specific to SRAs and decreased IFG activity could only be identified for NSRAs. The precuneus and posterior cingulate show elevated activity in SRAs, while findings regarding these areas were mixed in NSRAs. Additional scarce evidence suggests decreased ventral ACC activity and increased dorsal ACC activity in both addiction types. Associations between identified activation patterns with drug use severity underpin the clinical relevance of these findings. However, this exploratory evidence should be interpreted with caution and should be regarded as preliminary. Future research is needed to evaluate the findings gathered by this review.

**Supplementary Information:**

The online version contains supplementary material available at 10.1007/s11065-022-09552-5.

Addictive disorders are among the most common psychopathologies with lifetime prevalence rates ranging from around 1.2% for gambling disorder (American Psychiatric Association, 2013) to 9.9% for drug use disorder (Grant et al., 2016) and 21.9% for alcohol use disorder (Grant et al., 2015). Negative sequelae include considerable economic burden on society (Collins et al., 2000) and a reduced quality of life with elevated levels of mental and physical health problems in affected individuals (Bizzarri et al., 2005; Black et al., 2013). Addictive disorders encompass a broad spectrum of maladaptive behaviors, including substance-related addictions (SRAs) such as drug dependence and non-substance-related addictions (NSRAs) such as pathological gambling. A growing body of research shows that SRAs and NSRAs present similarities in behavioral as well as in biological alterations. Both, SRAs and NSRAs manifest similar core symptoms, such as withdrawal, tolerance, craving, and impaired behavioral control (Olsen, 2011; Potenza, 2008). Moreover, both addiction types are characterized by an inability to resist impulses towards rewarding stimuli despite potential adverse consequences (Potenza, 2006; West, 2001). The question as to why someone would repeatedly decide to engage in risky actions with possible adverse outcomes and despite significant negative consequences is puzzling. Greater insight into neural mechanisms of altered risk-taking behavior could contribute to a better understanding of maladaptive choice in addictive disorders.

Broadly, risk-taking is the tendency to choose an action with the potential for a relatively large, either beneficial or adverse outcome over an alternative that results in a comparably small, beneficial outcome. Thus, the individual has to evaluate the rewarding and punishing consequences of each option and their likelihood of occurring (Mellers et al., 1997; Paulus et al., 2003). Research on risk-taking has a long tradition and emerged predominantly from two fields. In economics, risk seeking is commonly defined as the preference for choosing an action with a higher, as opposed to lower, outcome variance holding the expected value constant (Hertwig et al., 2019; Schonberg et al., 2011). The outcome of such choices is probabilistic. In addition, the probability distributions of each possible outcome may be either known (decisions under risk) or unknown (decisions under uncertainty; Knight, 1921). Decisions which entail unknown probability distributions may best represent the psychological conception of risk, namely, behaviors with an uncertain potential for harmful outcomes for oneself or others (e.g., reckless driving, engaging in unprotected sex, free-climbing). Thus, in psychology, risky behavior is more broadly conceptualized as the tendency to engage in behaviors with potential detrimental consequences, but also with potential personal benefits, in situations which do not contain complete information about potential costs and benefits. This behavior is often seen in individuals with certain psychiatric disorders (e.g., Borderline Personality Disorder, Bipolar Disorder, Attention-Deficit/Hyperactivity Disorder). Addictive behaviors are also characterized by this kind of naturalistic risk-taking behavior from a psychological perspective: affected individuals show addictive behaviors despite potentially harmful, unknown consequences.

As a consequence of these different conceptualizations of risk-taking, different measurement traditions and paradigms have emerged and are used in neuroscientific studies investigating neural correlates of risk-taking and associated constructs. Insights gained through these studies point to several brain regions and networks involved. Early meta-analytic results identified several brain regions to be involved in the complex evaluation of decision alternatives (Krain et al., 2006; Mohr et al., 2010), including the dorsolateral prefrontal cortex (DLPFC), the ventromedial prefrontal cortex (vmPFC), the orbitofrontal cortex (OFC), the posterior parietal cortex (PPC), the anterior cingulate cortex (ACC), as well as the insula. Specifically, the vmPFC, the ventral striatum, and (in some cases) the posterior cingulate cortex are thought to form the core of the brain’s valuation system and to track the value of choice options across a variety of different contexts (Bartra et al., 2013; Lopez-Guzman et al., 2019). Activity in these brain areas can be observed both during decision-making and during outcome delivery (Bartra et al., 2013). In addition, activity in vmPFC and OFC is also associated with more emotional aspects of decision-making, such as affective attribution of choice (Ernst & Paulus, 2005), and it has also been shown to be involved in several other functions related to decision-making such as flexible representation of stimulus-outcome associations or inhibitory control (Murray & Rudebeck, 2018; Stalnaker et al., 2015). A more recent hypothesis proposes a unifying theory of OFC function which may integrate the above mentioned tasks, suggesting that the OFC may provide abstraction of currently available information/ higher-order representations that enable inference of unobservable information (Murray & Rudebeck, 2018; Stalnaker et al., 2015; Wilson et al., 2014; Yu et al., 2020). That is, it may represent the state of the world, all information that is relevant to the current decision. Thus, the OFC may provide a cognitive map of task/state space, a neural representation of stimuli, actions and other sensory features that occur in associations with outcomes.

The anterior insula and the (dorsal/caudal) striatum seem to be more related to emotional arousal or salience during risk-taking (Bartra et al., 2013). In addition, anterior insula activity codes individual risk perception (Christopoulos et al., 2009; Fukunaga et al., 2018; Paulus et al., 2003; Preuschoff et al., 2006). Another brain region that is commonly mentioned in the context of risk-taking is the DLPFC. It has been argued that during decision-making, it represents both cognitive and value-based information such as integrating rule-outcome associations and that it is especially activated during complex choices (Dixon & Christoff, 2014). The influence of the DLPFC on risk-taking may be of particular relevance for addictions due to its role in self-control.

Activity in many of these regions has been shown to differ in addiction. Moreover, research has already demonstrated that affective mechanisms such as reward processing and cognitive control (e.g., the inhibition of impulses) are altered in both SRAs and NSRAs. Specifically, altered (anticipatory) signaling in the amygdala and striatum seems to underlie aberrant reward processing in addictive disorders (in NSRAs as well as SRAs; Balodis & Potenza, 2015; García-García et al., 2014; Luijten et al., 2017). During inhibitory control tasks, individuals with addictive disorders show reduced activity in the ACC as well as prefrontal regions, including the DLPFC (Luijten et al., 2014). Since reward-processing as well as inhibitory control constitute essential and integral aspects of decision-making, it is likely that decision-making processes are also altered among individuals with addictive disorders (Balodis & Potenza, 2015; Hommer et al., 2011; Lawrence et al., 2009; Leyton & Vezina, 2013; Potenza et al., 2013).

A meta-analytic review from 2013 identified alterations in the ACC, OFC, DLPFC, striatum, insula, and the somatosensory cortex during risk-related decisions in individuals with SRAs (Gowin et al., 2013). Moreover, a sub-analysis of a recent meta-analysis on functional brain networks of different kinds of decision-making identified the thalamus, the caudate, and the cingulate to show reduced brain activity in SRAs (Poudel et al., 2020). It should be noted, however, that this meta-analysis included many studies employing paradigms which we do not consider in our review, such as the stop-signal task, which is an index for motor response inhibition rather than for decision making under risk or uncertainty. Moreover, the current literature is lacking an overview on risk-taking in individuals with NSRAs and comparisons between different addiction types.

The core of this comparative review is, therefore, to explore differences and similarities in neural correlates of risk-related decisions between SRAs and NSRAs. In NSRAs, phenotypic features and neural alterations are – in contrast to SRAs – not confounded by pharmacological effects of drugs, which cause additional neural changes (Goldstein & Volkow, 2011). Specifically, the objectives of this review are (1) to summarize neural correlates of risk-related decision-making in SRAs and NSRAs separately, and (2) to provide a preliminary investigation of commonalities and differences in neural correlates of risk-taking decision-making in individuals with SRAs and in individuals with NSRAs.

## Method

This review was not pre-registered and was conducted adhering to Preferred Reporting Items for Systematic Reviews and Meta-Analyses (PRISMA) guidelines (Mohr et al., 2010). A systematic literature search of the scientific databases PubMed and Web of Science, including all available empirical studies until January 2022, was conducted to find articles on functional neuroimaging studies investigating risky and uncertain decision-making in addictive disorders. Studies fulfilling inclusion criteria were identified by one author searching the databases after applying search terms, filters and exclusion criteria (see Table 1). In addition, reference lists of identified records were used to identify additional studies. Duplicates were removed, and titles and abstracts of remaining records were screened. In the present review, we consider studies investigating decision-making where outcome probabilities are tied to different choice options (i.e., are not random) and are either known (i.e., decisions under risk) or unknown (i.e., decisions under uncertainty) to participants. Finally, decisions should have rewarding or punishing consequences. We focused on statistical contrasts between risky decisions versus safe decisions or control conditions of the decision phase (in case where studies differentiated between different task phases). We included studies with a primary focus on whole-brain analyses (ROI analysis may be conducted in addition). Please refer to Table 1 for all inclusion criteria. For the ease of reading, we refer to both decision-making under risk and decision-making under uncertainty as risk-related decisions or risk-taking.

**Table 1:** PubMed and Web of Science electronic search and inclusion strategy for neural correlates of risk-related decision-making in addiction disorders

Search step	PubMed		Web of Science	
1	“"substance abuse" OR "substance use" OR "stimulant*" OR "cocaine" OR "heroin" OR "opiates" OR "opioids" OR "amphetamine" OR "methamphetamine" OR "cannabis" OR "marijuana" OR "smok*" OR "nicotine" OR "alcohol" OR "addiction" OR "gambling" OR "internet addiction" OR "internet gaming”		"substance abuse" OR "substance use" OR "stimulant*" OR "cocaine" OR "heroin" OR "opiates" OR "opioids" OR "amphetamine" OR "methamphetamine" OR "cannabis" OR "marijuana" OR "smok*" OR "nicotine" OR "alcohol" OR "addiction" OR "gambling" OR "internet addiction" OR "internet gaming"	
2	“decision making” OR “risk taking”	OR “Iowa Gambling Task” OR “Balloon Analog” OR “Balloon Analogue” OR “Wheel of Fortune”	“decision making” OR “risk taking”	OR “Iowa Gambling Task” OR “Balloon Analog” OR “Balloon Analogue” OR “Wheel of Fortune”
4	“fMRI” OR “PET”		“fMRI” OR “PET”	
5	Limit step 4 to language (English and German), and humans		Limit step 4 to language (English and German) and document type: articles	
6	Apply inclusion criteria (applied in the following order): 1. German or English language 2. Human subjects 3. Original research article (i.e., no review, meta-analysis) 4. Distinct addiction sample (studies including “occasional users” etc. were excluded) 5. Adult sample (≥ 18 years of age) 6. Control group (if no control group, within-group clinical variables, such as severity or duration of addiction should be investigated in relation to brain activation during risk-taking decision-making behavior) 7. Tasked-based fMRI/PET neuroimaging study (task of interest conducted during neuroimaging data acquisition) 8. Decision-making under risk or decision-making under uncertainty paradigm: - participants’ decisions should have rewarding or punishing consequences - outcome probabilities are tied to different choice options (i.e., are not random) 9. Primarily whole brain analyses (ROI analyses may be conducted in addition) 10. Risk contrasts: decision-phase: risky/disadvantageous decisions versus safe/advantageous decisions or control conditions			

Last search was conducted on 11.01.2022

## Results

**Fig. 1:**
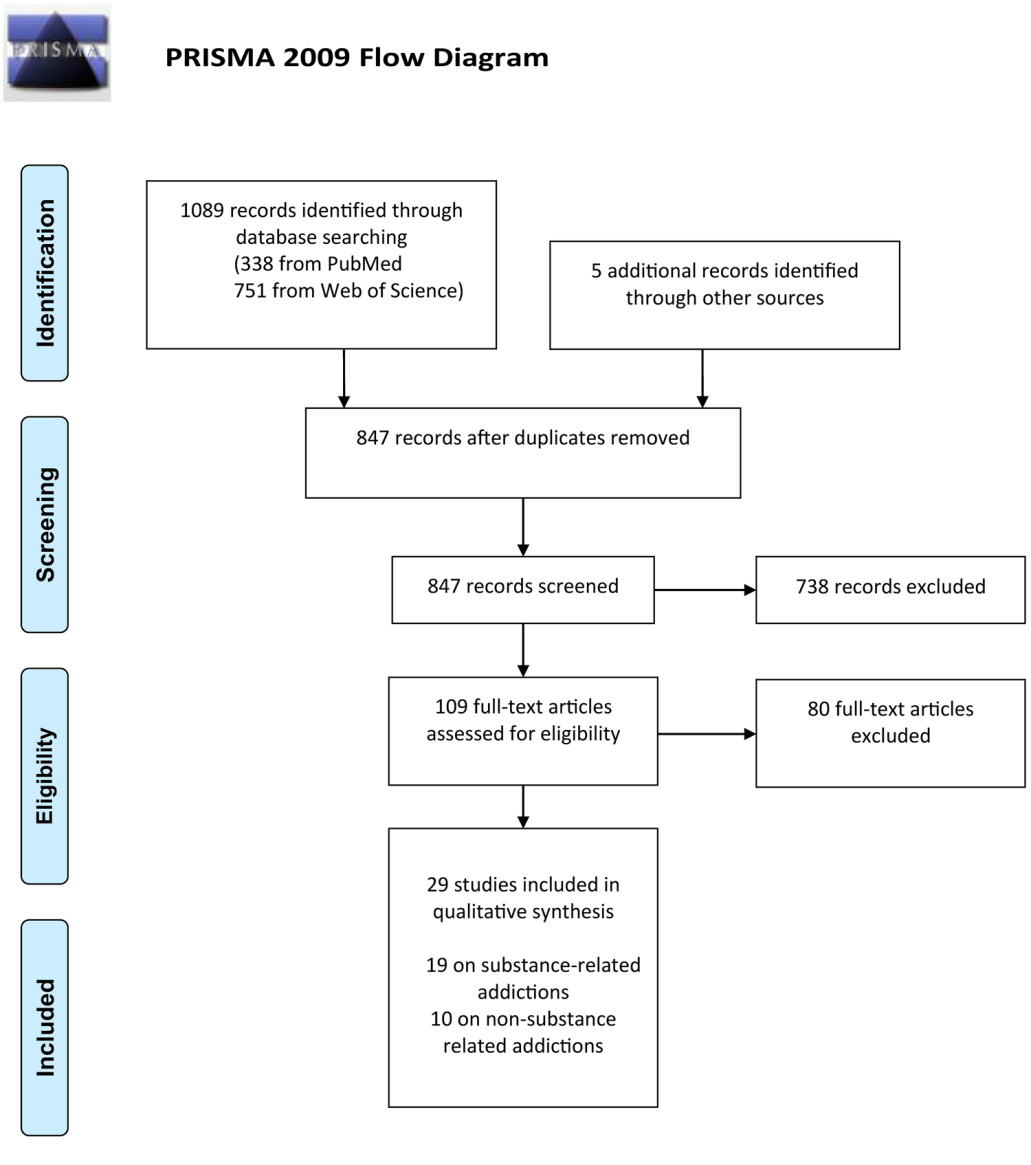
PRISMA flow diagram of study selection

A total of 847 studies were screened. After exclusion, 109 studies were assessed for eligibility. Finally, 29 studies examining neural correlates of risk-taking in addictive disorders were included and reviewed. Results of the study selection process are presented in Fig. 1. Included studies employed a variety of prominent risk-taking paradigms and five self-developed tasks (see Table S1 of the supplementary online material for an overview and description of the different tasks). In all paradigms, positive trial outcomes were associated with monetary wins or virtual points. Negative trial outcomes were associated with either a deduction of money/points or had no consequences. The risk contrasts of the decision phase (focus of the current review) of the imaging analyses aggregated over positive and negative trial outcomes of the subsequent outcome phase.

**Fig. 2:**
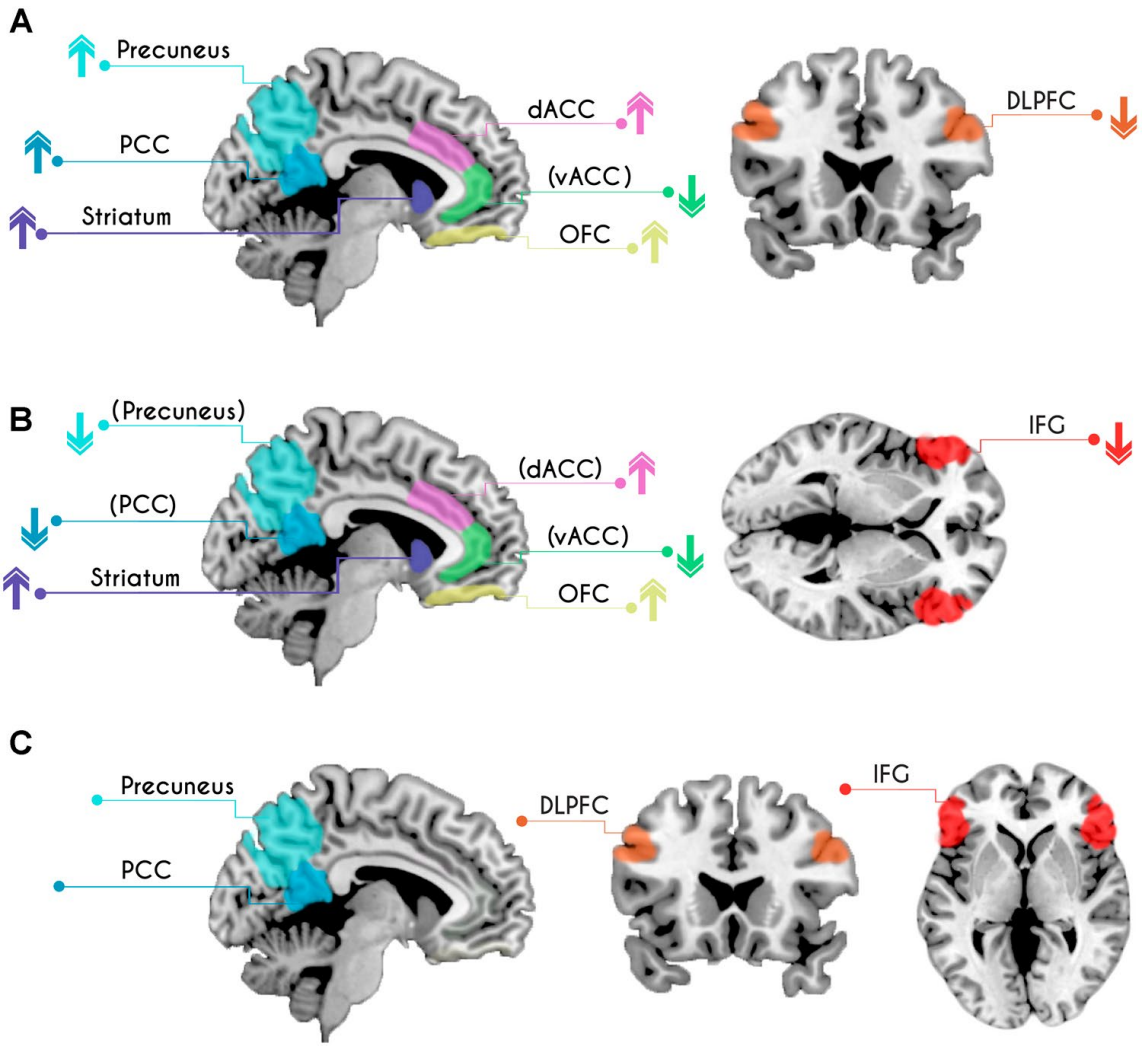
Altered risk-related brain activation patterns in individuals with **a** substance related addictions and **b** non-substance related addictions. Arrows indicate relative increase (↑) or decrease (↓) in brain activation compared to controls. **c** shows brain regions where substance related addictions and non-substance related addictions differ. Regions in brackets indicate mixed or limited evidence (< 3 studies reporting consistent alterations in this brain area)

Results of the present review are presented in two main sections. The first section presents findings on SRAs, and the second section presents findings on NSRAs which are compared to those of SRAs. In order to provide context, for each main section, we report findings on neural alterations in these groups stratified by studies reporting behavioral differences and studies which could not find any behavioral differences regarding risk-taking in comparison with controls. Each subsection is concluded by a brief summary of findings. Figure 2 compares findings on SRAs with those on NSRAs.

### Substance-related Addictions

Our review identified 19 eligible functional imaging studies which investigate risk-related decisions in SRAs compared to controls. A total of 648 individuals participated across all SRA studies. The number of included SRA individuals per study ranged from 11 to 75. Among the included studies, four studies were identified which did not include a healthy control group, but assessed the (longitudinal) relationship between clinical characteristics (e.g., symptom severity) and altered neural correlates during risk-taking (Blair et al., 2018; Forster et al., 2016, 2017; Gowin et al., 2014a). Approximately half (*k* = 7) of the reviewed studies examined risk-taking in individuals with stimulant addiction (*k* = 2 in cocaine addiction, *k* = 3 in methamphetamine addiction, and *k* = 2 in stimulant addiction, not further specified). Of the remaining studies, *k* = 3 studies investigated cannabis addiction, *k* = 2 studies investigated alcohol addiction and *k* = 5 studies did not report the exact addiction type or included mixed samples. Table 2 provides an overview of the study and sample characteristics of reviewed studies. Ten of the 19 studies reported increased risk-taking in individuals with SRAs, whereas nine of the 19 studies did not find any behavioral differences between individuals with SRAs and controls.

**Table 2:** Identified studies investigating neural correlates of risk-related decision-making in addiction disorders

**First author**	**n (male/female) population**	**Age (years ± SD or range)**	**Paradigm**	**Neuroimaging and analysis method**	**Brain (group) effects**	Behavioral (group) effects	Brain-symptom severity correlations
**Substance related addictions**							
Blair et al. (2018)	35 (16/19) STIM	20.74 ± 1.70 at baseline + 3 years follow-up	RGT (U)	fMRI	**Contrast: risk > safe** STIM > STIM desisted: cingulate, precuneus	Overall frequency of risky decisions: **Ø** Number of risky decisions after win: **↑** in STIM	n.a
	75 (46/29) STIM, desisted	20.95 ± 1.43 at baseline + 3 years follow-up					
Bolla et al. (2003)	13 (10/3) COC	36.1 ± 4.8	IGT (U)	PET: Whole brain ROI: OFC, DLPFC	**Contrast: decision making task > control task** COC > HC: r OFC (ROI), l postcentral gyrus, l putamen HC > COC: r DLPFC (ROI), l medial prefrontal cortex (ROI), r cerebellum, l medial frontal gyrus, l middle temporal gyrus, r superior parietal lobe	**Ø**	Weekly COC use **↑**: OFC **↓**
	13 (10/3) HCs	30.0 ± 6.3					
Bolla et al. (2005)	11 (11/0) CAN	21–35 (mean age: 26)	IGT (U)	PET: Whole brain ROI: OFC, DLPFC, hippocampus, cerebellum	**Contrast: decision making task > control task** CAN > HCs: l cerebellum (ROI), l parietal lobe: HCs > CAN: r OFC (ROI), r DLPFC (ROI)	**↑**	CAN use **↑:** cerebellum, orbital gyrus **↓**, parahippocampal gyrus **↑**
	11 (11/0) HCs	22–40 (mean age: 31)					
Burnette et al. (2020)	16 (11/5) ALC	31 ± 9.05	BART (U)	fMRI: Whole brain ROI: DLPFC	**Contrast: risk parametric regressor > control parametric regressor** HC > ALC: r frontal pole, r DLPFC (ROI)	**Ø**	n.a
	16 (11/5) HCs	30.94 ± 10.39					
Cousijn et al. (2013)	32 (26/15) CAN	21.4 ± 2.3 at baseline 21.9 ± 2.4 at six-month follow up	IGT (U)	fMRI: Whole brain	**Ø** at baseline	**Ø**	Longitudinal increase in weekly CAN use **↑:** bilateral lateral frontal pole, bilateral middle temporal gyrus, and bilateral inferior temporal gyrus **↑**
	41 (21/11) HCs	22.2 ± 2.4 at baseline 22.7 ± 2.4 at six-month follow up					
Ersche et al. (2005)	15 (14/1) AMP	37.6 ± 9.1	CRT (R)	PET: ROI: OFC, ACC, caudate/putamen, thalamus, ventral striatum, DLPFC, amygdala	**Contrast: decision making task > control task** AMP + OPI > HCs: b OFC (ROI) HCs > AMP + OPI: r DLPFC (ROI), l ACC (ROI)	**Ø**	n.a
	15 (15/0) OPI	37.1 ± 8.8					
	15 (9/6) HCs	35.8 ± 9.0					
Fishbein et al. (2005)	13 (7/6) SD	28.31 ± 5.19	CRT (R)	PET: ROI (30 different ROIs)	**Contrast: decision making task > control task** HCs > SD: perigenual/pregenual ACC (ROI)	**↑**	Risky decisions **↑:** insula **↑**, infragenual ACC, hippocampus, OFC, parietal lobe **↓** Symptom severity **↑**: OFC **↑**
	14 (7/7) HCs	25 ± 3.86					
Forster et al. (2016)	21 (15/6) SD	27 ± 6.8 at baseline + 3 months follow-up	BART (U)	fMRI: Whole brain correlational analyses	n.a	Longitudinal decrease in substance use **↓:** BART RT **↓**	Longitudinal decrease in substance use **↓:** putamen, middle temporal gyrus, dorsal ACC **↓**
Forster et al. (2017)	26 SD (17/9)	27 ± 6.5 at baseline + 3 months follow-up	BART (U)	fMRI: Whole brain correlational analyses	n.a	Longitudinal increase in substance use **↑**: higher number of risky choices (inflations on the BART) **↑**	Longitudinal increase in substance use **↑**: l/r supplementary motor area **↑**
Fukunaga et al. (2013)	22 (10/12) SD	21.5 ± 0.5	IGT (U)	fMRI: Whole brain	**Contrast: risky decisions > safe decisions** HCs > SD: ACC/DMPFC, insula	**↑**	
	25 (14/11)HCs	22.3 ± 0.6					
Gilman et al. (2015)	18 (12/6) ALC	31.2 ± 7.1	Lane Risk-Taking Task (U)	fMRI: Whole brain	**Contrast: cue risky > cue safe** HCs > ALC: l DLPFC/superior frontal	**Ø**	Risky choice modulated by earnings **↑:** middle frontal gyrus, precuneus, posterior cingulate, thalamus, putamen **↑**
	18 (12/6) HCs	30.5 ± 5.1					
Gowin et al. (2014a)	18 METH relapsed	37.4 ± 9.2	RGT (U)	fMRI: Whole brain ROI: ACC, insula	**Contrast: risk > safe** METH abstinent > METH relapsed: insula (ROI)	**Ø**	Risky decisions **↓**: anterior insula **↑**
	45 METH abstinent	38.8 ± 11.1					
Gowin et al. (2014b)	68 (53/15) METH	38.2 ± 10.5	RGT (U)	fMRI: Whole brain ROI: ACC, insula	**Contrast: risk > safe** METH > HCs: dorsal ACC (ROI), insula (ROI)	Overall frequency of risky decisions: **Ø** Risky decisions after losses: **↑**	Use history **↑**: rostral ACC, mid insula Frequency of risky choice post losses: rostral ACC **↓**, mid-insula **↑**
	40 (26/14) HCs	35.6 ± 11.5					
Gowin et al. (2017)	32 (28/4) COC	44.21 ± 9.16	RGT (U)	fMRI: Whole brain ROI: striatum, insula, ACC	**Contrast: risk > safe** HCs > COC: ventral striatum (caudate), ventral striatum (ROI) COC > HCs: dorsal ACC (ROI)	Overall frequency of risky decisions: **Ø** Risky decisions after losses: **↑**	Lifetime cocaine use **↑**: dorso-rostal ACC **↑** Time since last use **↑**: dorsal ACC **↑**
	40 (26/14) HCs	35.60 ± 11.58					
Kohno et al. (2014)	25 (12/13) METH	35.68 ± 1.64	BART (U)	fMRI: Whole brain ROI: right DLPFC, striatum	**Contrast: risk parametric regressor > control parametric regressor** METH > HCs: b ventral striatum (ROI) HCs > METH: r DLPFC (ROI and whole brain)	**Ø**	
	27 (16/11) HCs	33.88 ± 2.3					
Tanabe et al. (2007)^a^	20 (10/10) SD	35 ± 7	IGT (U)	fMRI: Whole brain	**Contrast: risky decisions > safe decisions** H SDPG/SD > HCs: l Ventral medial frontal cortex	**Ø**	n.a
	20 (12/8) SDPG	35 ± 7					
	16 (5/11) HCs	37 ± 9					
Vaidya et al. (2012)	46 (28/18) CAN	24.32 ± 3.85	IGT (U)	PET: Whole brain ROI: vmPFC	**Contrast: decision making task > control task** CAN > HCs: r cerebellum, r vmPFC (ROI) HCs > CAN: l superior temporal gyrus	For regular IGT: **Ø**	duration of use **↑:** vmPFC **↑**, dorsal ACC **↓**
	34 (18/16) HCs	24.72 ± 5.25					
Yamamoto et al. (2014)	37 (18/19) STIM	34.4 ± 8.4	IGT (U)	fMRI: Whole brain ROI: ventral striatum, dorsal striatum, OFC, insula, ACC	**Contrast: decision making trials > control trials** STIM > HCs: l thalamus/striatum, r pallidum, l precuneus, r middle cingulate, r ACC, r inferior parietal, l cerebellum, r superior parietal, r middle frontal, r superior temporal, r postcentral, r medial superior frontal, r medial frontal, l cuneus, r inferior orbital, r insula, dorsal striatum (ROI), ventral striatum (ROI), insula (ROI), ACC (ROI), OFC (ROI)	**↑**	n.a
	43 (23/20) HCs	31.6 ± 9.3					
Yamamoto et al. (2017)	25 (14/11) STIM	35.2 ± 6.9	Modified IGT (U)	fMRI: Whole brain ROI: OFC, DLPFC	**Contrast: decision making trials > control trials** STIM > HCs: l OFC (ROI) Ø for whole-brain	**↑**	n.a
	20 (11/9) HCs	33.2 ± 8.6					
**Non-substance related addictions**							
Brevers et al. (2015)	12 (8/2) PG	34.00 ± 8.53	Modified Card Deck Paradigm (R/U)	fMRI: Whole brain	**Contrast: bet choice > safe choice** PG > HCs: r putamen	**↑**	n.a
	12 (8/2) HCs	36.20 ± 12.95					
Dong and Potenza (2016)	20 (20/0) IGD	21.33 ± 2.18	Self-developed task (R/U)	fMRI: Whole brain	**Contrast: risk disadvantageous > risk advantageous** HCs > IGD: l posterior cingulate cortex, l ACC, r middle temporal gyrus, l superior temporal gyrus, l cuneus, l inferior parietal cortex, l inferior frontal cortex	**↑**	n.a
	16 (16/0) HCs	21.9 ± 2.33					
Lin et al. (2015)	19 (19/0) IGD	22.2 ± 3.08	PDT (R)	fMRI: Whole brain	**Contrast: probabilistic option > safe option** HC > IGD: l inferior frontal gyrus, l precentral gyrus	**↑**	n.a
	27 (27/0) HCs	22.74 ± 2.35					
Liu et al. (2017)	41 (41/0) IGD	21.93 ± 1.88	Cups Task (R)	fMRI: Whole brain	**Contrast: risky choices > safe choices (loss domain)** IGD > HCs: l insula/OFC: **↑** **Contrast: parametric risk** **(loss domain)** HCs > IGD: l middle frontal gyrus/ inferior frontal gyrus, r inferior occipital gyrus, r inferior parietal/precuneus	**Ø**	Severity of IGD **↑:** DLPFC, inferior parietal lobe **↓**
	27 (27/0) HCs	22.74 ± 2.35					
Miedl et al. (2010)	12 (12/0) PG	39.5 ± 9.3	Blackjack Game (U)	fMRI: Whole brain	**Contrast: high risk > low risk** PG > OG: r inferior frontal gyrus, r superior temporal gyrus, bilateral thalamus	**Ø**	n.a
	12 (12/0) Occasional gamblers	33.4 ± 8.0					
Miedl et al. (2014)	4 Problem gamblers (4/0) 8 PG (8/0)	33.8 ± 7.8	Blackjack Game (U)	Combined EEG/fMRI	**Contrast: high risk > low risk** (at 600–800 ms post-stimulus onset) PG > OG: r thalamus, l OFC, l superior frontal gyrus	**Ø**	n.a
	12 (12/0) Occasional gamblers	35.8 ± 9.5					
Power et al. (2012)	13 (13/0) PG	42.4 ± 10.8	IGT (U)	fMRI: Whole brain	**Contrast: high risk > low risk** PG > HCs: r caudate nucleus, r OFC, r amygdala, r superior frontal gyrus, brainstem HCs > PG: r occipital, r occipital fusiform	Over last three quartiles: **↑**	n.a
	13 (13/0) HCs	41.0 ± 11.0					
Seok et al. (2015)	15 (15/0) IGD	22.20 ± 3.07	PDT (R)	fMRI: Whole brain ROI: dorsal ACC, caudate	**Contrast: risky option > safe option** IGD > HCs: l caudate nucleus, l dorsal ACC HCs > IGD: l vlPFC	**↑**	n.a
	15 (15/0) HCs	22.47 ± 2.53					
Wang et al. (2017)	20 (20/0) IGD	20.95 ± 2.44	PDT (R)	fMRI: Whole brain	**Contrast: probabilistic option > safe option** HCs > IGD: l inferior frontal gyrus, l precentral gyrus, r medial frontal gyrus	**↑**	n.a
	20 (20/0) HCs	21.95 ± 2.37					
Wang et al. (2021)	27 (NA/NA) IGD	22.52 ± 2.33	PDT (R)	fMRI: Whole brain ROI: clusters that survived the threshold of voxel-level uncorrected Z > 2.58 were defined as ROIs	**Contrast: bet > safe option** IGD > HCs: r nucleus accumbens ROI: IGD > HCs: r nucleus accumbens, l middle occipital gyrus, l precuneus, l middle temporal gyrus	**↑**	n.a
	26 (NA/NA) HCs	23.23 ± 2.37					

Labels of brain regions as reported in the original studies

*SD* Standard deviation, *ROI* region of interest, **↑** increased, **↓** decreased, *PET* positron-emission tomography, *fMRI* functional magnetic resonance imaging, *n.a.* not available, *EEG* electroencephalography, *U* uncertainty task, *R* risk task, *ALC* alcohol, *AMP* amphetamine, *CAN* cannabis, *COC* cocaine, *IGD* internet gaming disorder, *HCs* healthy controls, *METH* methamphetamine, *OPI* opiates, *PG* pathological gamblers, *STIM* stimulants, *SDPG* substance dependent pathological gamblers, *SU* substance user (sample included various different user classes), *BART* Balloon Analogue Risk Task, *CRT* Cambridge Risk Task, *IGT* Iowa Gambling Task, *PDT* Probability Discounting Task, *RGT* Risky Gains Task, *r* right, *l* left, *b* bilateral, *ACC* anterior cingulate cortex, *DLPFC* dorsolateral prefrontal cortex, *DMPFC* dorso medial prefrontal cortext, *OFC* orbitofrontal cortex, *vlPFC* ventrolateral prefrontal cortex, *vmPFC* ventro medial prefrontal cortex

^a^This study included individuals with substance-related addictions as well as non-substance related addictions

#### DLPFC and Extending Regions

Across different tasks and decision-making domains, activation within prefrontal areas, particularly within the right DLPFC (middle frontal gyrus) was decreased in individuals with SRAs (*k* = 6; Bolla et al., 2003; Bolla et al., 2005; Burnette et al., 2020; Ersche et al., 2005; Gilman et al., 2015; Kohno et al., 2014). Other prefrontal areas which were reported to show decreased risk-related brain activity in individuals with SRAs included the superior and medial frontal gyri (Bolla et al., 2003; Burnette et al., 2020; Gilman et al., 2015). Among the six studies reporting decreased risk-related DLPFC activity, only one study found increased behavioral risk-taking in the SRA group; the remaining studies did not find any behavioral differences between SRAs and HCs. One study reported increased middle frontal (DLPFC), and medial/superior frontal activity in individuals with SRAs (Yamamoto et al., 2014). Finally, a longitudinal study on SRA investigated substance use outcomes for a duration of three months and reported a positive correlation between substance use and activation in the supplementary motor area on the BART task (Forster et al., 2017).

#### ACC

Three studies reported a trend toward riskier behavior in individuals with SRAs on an uncertainty task (specifically, an increased frequency of risky decisions after losses) and also identified increased risk-related dorsal ACC activity (*k* = 3; Gowin et al., 2017, 2014b; Yamamoto et al., 2014). Interestingly, the studies conducted by Gowin et al. revealed that their control participants showed greater dorsal ACC activity during safe relative to risky choices whereas their SRAs groups either showed enhanced activity when they chose risky options or showed no differential activity for risky compared to safe choices. In addition, increased risk-related dorsal ACC activity seems to be related to greater symptom severity (Gowin et al., 2017). This finding is supported by a longitudinal study which tracked substance use in an SRA sample throughout a 3-month period. Improved substance use outcomes were related to a decreased risk-related response in the dorsal ACC (extending to the vmPFC; as well as in the putamen) at follow-up relative to a baseline assessment (Forster et al., 2016). Conversely, duration of use was negatively correlated with dorsal ACC activity (Vaidya et al., 2012).

Conversely, the ventral ACC showed decreased risk-related activity in the context of similar (Ersche et al., 2005) as well as increased behavioral risk-taking (Fishbein et al., 2005) in SRAs compared to HCs (*k* = 2). Both studies used the Cambridge Risk Task which measures decision-making under risk.

#### Insula/IFG

In total, *k* = 2 studies reported decreased risk-related brain activity in the anterior insula under uncertainty. Specifically, relapsed as opposed to abstinent individuals with SRAs showed blunted risk-related activity in the anterior insula (Gowin et al., 2014a). Furthermore, Fukunaga et al. (2013) identified regions which showed a difference in brain activation associated with risky relative to safe decisions. They further investigated regions showing such a difference and found that individuals with SRAs showed lower differential activity in the anterior insula that corresponded to greater risk-taking behavior.

Conversely, risk-related activity in the insula/mid insula was found to be increased in SRAs under uncertainty (*k* = 2; Gowin et al., 2014b; Yamamoto et al., 2014). Increased activity in the mid insula correlated with the frequency of risky choices after losses as well as with a longer substance use history (Gowin et al., 2014b).

#### OFC/vmPFC

Regions around the OFC/vmPFC were reported to exhibit increased risk-related activity in SRAs (*k* = 6; Bolla et al., 2003; Ersche et al., 2005; Tanabe et al., 2007; Vaidya et al., 2012; Yamamoto et al., 2014, 2017). Moreover, a positive relationship between risk-related OFC activity and duration of substance use (Vaidya et al., 2012) as well as symptom severity was reported (Fishbein et al., 2005). Of those, one study employed a decision-making under risk task, whereas the remaining studies employed the IGT (decision-making under uncertainty). Only one of the five studies that reported increased OFC activity also identified increased behavioral risk-taking in SRAs (during the IGT). A further study which found elevated levels of behavioral risk-taking (also employing the IGT), revealed decreased risk-related OFC activity in individuals with SRAs (Bolla et al., 2005).

#### Striatum

The ventral striatum as well as the putamen showed increased risk-related activity in SRAs in two studies where behavioral performance did not differ between SRAs and HCs under uncertainty (Bolla et al., 2003; Kohno et al., 2014). In addition, Yamamoto et al. (2014) found increased risk-related activity in the dorsal and ventral striatum, extending to the thalamus and the pallidum, paralleled by increased risk-taking in SRAs on the IGT. Thus, in total, *k* = 3 studies reported increased striatal activity in SRAs. Conversely, one study found decreased ventral striatal activity (caudate) in a sample of individuals with SRAs who exhibited increased risk-taking behavior after experiencing losses under uncertainty (*k* = 1; Gowin et al., 2017). The putamen seems to respond particularly in the context of rewards as shown by Gilman et al. (2015). These authors investigated the effect of accumulated rewards on risk-related brain activation. Specifically, they found that individuals with SRAs exhibit heightened activation in the putamen (in addition to the posterior cingulate, the precuneus, and the thalamus) that correlated with the total accumulated reward during decisions under uncertainty (Gilman et al., 2015). Correspondingly, a longitudinal study on individuals with SRAs reported decreased putamen (as well as decreased dorsal ACC) activity at follow-up relative to a baseline assessment to correlate with less substance use during the study interval (Forster et al., 2016).

#### Parietal Lobe

In total, *k* = 3 studies revealed increased risk-related activity of the postcentral gyrus/posterior parietal lobe under uncertainty (Bolla et al., 2003, 2005; Yamamoto et al., 2014). Two of those, also reported increased behavioral risk-taking in the SRA group, while the other study found no elevated risk-taking. In addition, Yamamoto et al. (2014) reported increased superior parietal activity, while Bolla et al. (2003) found decreased activity in the superior parietal lobe in their SRA group. The precuneus, a neighboring region of the superior parietal lobe also seems to be involved in risk processing. One study reported increased risk-related precuneus activity in SRAs compared to controls (Yamamoto et al., 2014). In addition, Blair et al. (2018) conducted a longitudinal study on stimulant users and investigated whether risk-related brain activity differentiated occasional stimulant users who became problem stimulant users from those who desisted from stimulant use. Those who became problem users showed greater precuneus and (posterior cingulate) activity during risk-related decisions as opposed to those participants who desisted from use. Similarly, Gilman et al. (2015) reported increased activation in the precuneus (in addition to the posterior cingulate, the thalamus, and the putamen) that correlated with total accumulated reward during risk-taking under uncertainty (Gilman et al., 2015).

#### Temporal Lobe

Three (decision-making under uncertainty) studies that included a HC group reported risk-related alterations in the temporal lobe in SRAs. All used the IGT task. Alterations included decreased activity in the middle temporal gyrus (Bolla et al., 2003), and decreased (Vaidya et al., 2012) as well as increased activity in the superior temporal gyrus (Yamamoto et al., 2014). A longitudinal study on a SRA-only group reported increased risk-related activity in the middle temporal as well as inferior temporal gyrus on the IGT to be associated with an increase in substance use after 6 months (Cousijn et al., 2013). Similarly, another longitudinal study on individuals with SRAs found a longitudinal decrease in substance use over a 3-month period to correlate with less activity in the middle temporal gyrus (Forster et al., 2016). Both longitudinal studies used decision-making under uncertainty tasks.

#### Additional Areas

One study reported increased risk-related activity in the cuneus (Yamamoto et al., 2014) and *k* = 4 studies identified altered activity in the cerebellum on the IGT. Of those, three studies reported increased cerebellar activity (Bolla et al., 2005; Vaidya et al., 2012; Yamamoto et al., 2014) and one reported decreased cerebellar activity (Bolla et al., 2003).

#### Summary

Among studies which investigated risk-related decision-making in SRAs, convergent attenuated brain activity was reported for prefrontal areas, especially the right DLPFC. This finding seems to be independent of risk-taking behavior, as it was reported by studies which observed behavioral differences in risk-related decision-making and studies which did not observe such differences. Results further point to a hyperactivation of the OFC/vmPFC and associations between OFC hyperactivity and symptom severity across several studies. Moreover, some evidence suggests risk-related alterations in additional brain areas, including the posterior cingulate cortex, the precuneus and the postcentral gyrus which show increased activity across studies, and also show associations to reward. While results point to decreased risk-related activity in the anterior insula, risk-related activity may be increased in the mid insula. Results regarding the (dorsal) striatum and the dorsal ACC point to elevated risk-related brain activity. Increased dorsal ACC activity was mostly reported by studies which could also show trends towards increased behavioral risk-taking or could show associations to clinical symptoms. In contrast, activity in the ventral ACC may be decreased in SRAs. Findings on the temporal lobe are mixed but point to a positive relationship between activity in the middle temporal lobe and a longitudinal increase in substance use.

### Non-substance Related Addictions

In total, 10 studies investigated neural correlates of risk-related decisions in individuals with NSRAs, with *k* = 6 studies on individuals with Internet Gaming Disorder (IGD) and *k* = 4 studies on Pathological Gambling. Of those, seven studies did find elevated levels of behavioral risk-taking in individuals with NSRAs, whereas three studies could not find any behavioral differences regarding risk-taking behavior in NSRAs relative to controls. A total of 187 individuals participated across all NSRA studies. The number of included NSRA individuals per study ranged from 8 to 41. Table 2 provides an overview of study and sample characteristics of reviewed studies. No symptom-severity-relationships were reported by any of these studies.

#### DLPFC and Extending Regions

Decreased risk-related brain activity in the DLPFC could only be identified by one study (Liu et al., 2017). In this study, participants chose between a safe and a risky option (decision-making under risk task), either in a gain domain, where they should win as much money as possible or in a loss domain, where they should avoid losing money. Parametric analyses revealed decreased activation in the DLPFC (and the inferior parietal lobe/precuneus) in individuals with Internet Gaming Disorder compared to controls – only for the loss domain. No group differences were found for the gain domain. It should be noted that all other studies reviewed here employed tasks, where participants played for potential gains. In addition, decreased frontal activity was reported for the medial frontal gyrus (*k* = 1; Wang et al., 2017) as well as the precentral gyrus (*k* = 2; Lin et al., 2015; Wang et al., 2017). These studies revealed increased risk-taking behavior in individuals with NSRAs on probability discounting tasks (decision-making under risk).

The superior frontal gyrus was reported to show increased risk-related activity in *k* = 2 decision-making under uncertainty studies. Increased risk-taking in NSRAs was reported only by one of these studies (Power et al., 2012) but not by the other (Miedl et al., 2014).

#### ACC

Decreased brain activation under risk was reported for the ventral ACC which was accompanied by increased behavioral risk-taking in individuals with NSRAs (*k* = 1; Dong & Potenza, 2016).

In contrast, increased brain activation was identified for the dorsal ACC during a decision-making under risk task (*k* = 1; Seok et al., 2015). This study also revealed increased risk-taking behavior.

#### Insula/IFG

A total of *k* = 5 studies identified decreased risk-related activity in the left IFG/ventrolateral prefrontal cortex in NSRAs (Dong & Potenza, 2016; Lin et al., 2015; Liu et al., 2017; Seok et al., 2015; Wang et al., 2017). All, but one study used decision-making under risk tasks and revealed increased behavioral risk-taking in individuals with NSRAs. The study which could not reveal increased risk-taking employed the Cups Task (decision making under risk) and – as mentioned before – reported effects for conditions focusing on loss avoidance (Liu et al., 2017). In contrast, in a decision-making under uncertainty setting which did not reveal any behavioral differences between individuals with NSRAs and HCs, right IFG activity seems to be increased (*k* = 1; Miedl et al., 2010).

#### OFC/vmPFC

In total, *k* = 3 studies (*k* = 2 decision-making under uncertainty and *k* = 1 decision-making under risk) revealed increased OFC/vmPFC activity in individuals with NSRAs. Of those, two studies investigated decision-making under uncertainty (Miedl et al., 2014; Power et al., 2012) and one study investigated decision-making under risk (Liu et al., 2017).

#### Striatum

Increased striatal activity in individuals with NSRAs was reported by *k* = 4 studies. All of them also reported increased risk-taking in NSRAs. The dorsal striatum (putamen and well as caudate nucleus) showed increased activity in *k* = 3 studies (under risk as well as uncertainty; Brevers et al., 2015; Power et al., 2012; Seok et al., 2015). Moreover, the adjacent nucleus accumbens (ventral striatum) showed increased activity under risky decision-making (*k* = 1; Wang et al., 2021).

#### Parietal Lobe

The inferior parietal lobe/the precuneus showed reduced activity in *k* = 2 studies (Dong & Potenza, 2016; Liu et al., 2017). Specifically, Liu et al. (2017) could not identify any behavioral alterations during a decision-making under risk task in their NRA group. Moreover, as mentioned above, they could not find any group differences when their participants played for potential gains – only when they played to avoid losses. Dong and Potenza (2016) reported less activation in the inferior partial lobe (and the posterior cingulate cortex) along with increased risk-taking behavior in individuals with NSRAs during a decision-making under uncertainty task. In contrast, one study identified increased precuneus activity and increased risk-taking behavior under risky decision-making (Wang et al., 2021).

#### Temporal Lobe

Findings on the temporal lobe are mixed. In total, *k* = 3 studies reported altered risk-related temporal activity. Specifically, Dong and Potenza (2016) reported decreased superior temporal and middle temporal activity (accompanied by increased behavioral risk-taking). In contrast, Miedl et al. (2010) reported increased superior temporal activity and Wang et al. (2021) reported increased middle temporal activity.

#### Additional Areas

In addition, *k* = 2 studies identified increased risk-related activity in the thalamus during decision-making under uncertainty (Miedl et al., 2010, 2014). Power et al. (2012) employed the IGT (decision-making under uncertainty) and reported increased activity in the amygdala and in the brainstem which was accompanied by increased risk-taking. Finally, several studies (*k* = 3) reported decreased activity in occipital brain regions for decisions under risk (*k* = 2; Dong & Potenza, 2016; Liu et al., 2017) and under uncertainty (*k* = 1; Power et al., 2012). In contrast, one study found increased activity in the middle occipital gyrus under risk (Wang et al., 2021).

#### Summary

Taken together, studies on risk-related decision-making in individuals with NSRAs reported widespread activation differences compared to controls. Concordant alterations point to hypoactivity in the inferior frontal cortex and hyperactivity in the OFC/vmPFC in both those studies reporting increased behavioral risk-taking in NSRAs and those studies which did not report such behavioral differences. In contrast, (dorsal) striatal hyperactivity was only reported by studies that also found increased behavioral risk-taking. Additional preliminary evidence points to hyperactivity of the thalamus, the dorsal ACC and hypoactivity of the ventral ACC. Findings regarding further brain areas are rather mixed.

Compared to studies on SRAs, this review identified both overlapping and specific brain activation patterns for NSRAs. These preliminary findings suggest concordant alterations in SRAs and NSRAs in the OFC/vmPFC and the striatum. These regions show increased activity in both addiction types during risk-related decisions. Across studies, increased activity could be identified in both SRAs and NSRAs, irrespective of behavioral task performance. Increased dorsal ACC activity was also reported for both addiction types, but only in studies which also revealed relationships to behavioral risk-taking. In contrast, studies that identified neural alterations in the ventral ACC revealed decreased activity in both addiction groups. Interestingly, the postcentral gyrus, posterior cingulate and precuneus activity seem to be increased in SRAs. In contrast, activity in these regions seem to be decreased in NSRAs. We could also identify differing risk-related patterns of brain activity for the two addiction types. A finding specific to SRA studies is decreased right DLPFC activity, whereas left IFG activity was mostly reported by NSRA studies. To clarify if frontal areas that altered in NSRAs are distinct from the DLPFC, we extracted reported brain coordinates and overlaid them with a DLPFC mask (see supporting information Fig. S1). This underlined that reduced DLPFC activity may only occur in individuals with SRAs.

## Discussion

We here examined neural correlates of risk-related decision making in individuals with SRAs and individuals with NSRAs. Furthermore, we aimed to provide preliminary comparative results of these two groups to identify overlapping as well as differing alterations in neural correlates of risk-related decision-making. This approach is in line with the Research Domain Criteria principle of studying mental disorders beyond their diagnostic boundaries (Cuthbert & Insel, 2013).

Although currently available studies on risk-related decision-making, especially in the domain of NSRAs, are sparse, we did find interesting similarities and differences between the two addiction types that may be further validated by future studies. One difference relates to alterations in posterior cingulate and precuneus activation. These areas may be affected by both SRAs and NSRAs. However, while activity in these regions seems to be increased in SRAs, the activity appears to be decreased in NSRAs. Activity in these regions in response to risky situations has also been observed in normative samples (Paulus et al., 2003; Roy et al., 2011) and it has been suggested that these regions may be involved in tracking risky subjective value and may provide a willingness to accept uncertain outcomes for a chance to receive greater rewards (Coutlee et al., 2016). Interestingly, decreased precuneus/inferior parietal lobe activation in NSRAs was reported, amongst others, for situations where participants played to avoid losses. This experimental situation differs from all other studies reviewed here, which all employed tasks where participants play for potential gains. Future studies may ascertain whether individuals with SRAs also show increased precuneus/inferior parietal lobe activation under loss avoidance situations. The precuneus and the posterior cingulate cortex are part of the default mode network, which is active during self-referential processes, such as self-awareness and self-reflection (Andrews-Hanna, 2012). A review on the involvement of different brain networks in different addiction cycles concluded that default mode network activity was decreased during decision-making tasks in individuals with SRAs (Zilverstand et al., 2018). This stands in contrast to results of our review suggesting increased default mode network activity. It should be noted that the review by Zilverstand et al. (2018) included studies which focus on outcome-processing (i.e., loss evaluation), whereas our review focuses on the actual decision stage. Together with our findings on NSRAs, it may be speculated that default mode network activity may be increased during risk-related decisions and decreased under loss evaluation conditions in addiction. However, this speculation needs to be further investigated.

Another area of divergence relates to the inferior frontal gyrus (IFG). Compared to HCs, neural activity during risk-taking in this brain region is reduced in individuals with NSRAs. Our systematic review could not find any evidence for altered patterns of IFG activity in SRAs. However, we did find reduced activity in the anterior insula, a region adjacent to the IFG, in SRAs. The anterior insula is often coactivated with the IFG and reviewed studies may have detected and reported peak activation only for one of the regions. Activity in the IFG/anterior insula is modulated by individual risk processing and more specifically by risk/harm avoidance (Christopoulos et al., 2009; Fukunaga et al., 2018; Paulus et al., 2003; Preuschoff et al., 2006). Interestingly, studies that reported diminished IFG activity in NSRAs all used decision-making under risk tasks and reported increased risk-taking behavior. The majority of studies on SRAs employed decision-making under uncertainty tasks and, mostly, could not reveal altered risk-taking behavior. One of the reviewed studies that reported diminished activity in the anterior insula, extending to the IFG also found increased risk-taking behavior in their SRA group. In fact, they reported a link between risk behavior and anterior insula activity where task performance of the SRA group reflects lower risk-aversion signals in the anterior insula/IFG (Fukunaga et al., 2013). The extent to which diminished IFG activity in NSRAs may relate to task properties, risk perception, or another factor specific to individuals with NSRAs remains to be further investigated.

The dorsal ACC is closely connected to the anterior insula/IFG and is also implicated in a wealth of functions during decision-making, such as signaling error and reward (Alexander & Brown, 2019), as well as the value of behavioral change and individuals risk tendencies (Fukunaga et al., 2018; Kolling et al., 2012, 2016). For both addiction types, findings regarding the dorsal ACC point to increased activity, which was always accompanied by increased behavioral risk-taking. As suggested elsewhere, for SRAs the dorsal ACC may fail to integrate outcomes into an online prediction of what will happen for each choice, which leads to repeatedly choosing disadvantageous options (Gowin et al., 2017). Additionally, increased dorsal ACC activity may be related to symptom severity in SRAs.

Another difference in findings regards the DLPFC. Hypoactivation of the right DLPFC was characteristic of SRAs during risk-related decisions across a variety of different tasks. DLPFC hypoactivation in SRAs has been identified before in previous reviews (Gowin et al., 2013) and it has been suggested that structural and functional alterations in the DLPFC may originate from severe drug use (Bolla et al., 2005). This is supported by structural imaging studies reporting reduced prefrontal grey matter density in several SRAs, including alcohol, cocaine, methamphetamine, and heroin addiction (Goldstein & Volkow, 2011). The right DLPFC plays a rather specific role in risk-related decision-making and is involved in valuing and integrating choice options before making a decision (Ernst & Paulus, 2005; Mohr et al., 2010). Moreover, it has been argued that DLPFC activity during value-based processes cannot be attributed entirely to a strict “cognitive” role. In fact, it may represent value information in relation to cognitive information (e.g., task-rules) providing a top-down control on core value regions (Dixon & Christoff, 2014). Interestingly, areas belonging to the brain’s valuation system such as the vmPFC, the striatum as well as the posterior cingulate show increased brain activation in studies reporting decreased DLPFC activity (c.f. Table 2), supporting the notion of a potential top-down influence of the DLPFC on the value system. However, this is an associative observation and may be tested by connectivity analyses. Attenuated risk-related activity in the DLPFC may thus lead to a devaluation of disadvantageous stimuli during decision-making in SRAs and to a reduced top-down influence on core value regions. We could only identify sparse evidence for reduced risk related (right) DLPFC activity in NSRAs. Due to paucity of data, firm conclusions, however, cannot be drawn about functional DLFPC reactivity in response to risk-related decision-making in NSRAs. Further research is needed in order to clarify whether individuals with NSRAs are able to recruit the DLPFC during risk-related decision making or not.

As mentioned above, results of the current review point to increased striatal as well as OFC activity under risky situations in SRAs but also in NSRAs. Areas adjacent to the striatum such as the thalamus and the globus pallidus also seem to show increased risk-related activity as reported by some studies. These findings represent a similarity across addiction types and suggest that alterations in these brain areas may be implicated in the psychopathology of addictive disorders. The striatum and the vmPFC have been ascribed to the brain’s core value system. In the neuroscientific literature, it is well established that they represent reward and preference and automatically track subjective value across different contexts (Bartra et al., 2013; Clithero & Rangel, 2014; Levy & Glimcher, 2012; Schmidt et al., 2017). In addition to its role in tracking subjective value during value-based decisions, the striatum (especially the dorsal striatum) has been associated with action-contingent learning in addiction and has been implicated in the transition from recreational to chronic use (Everitt & Robbins, 2013). Moreover, frontostriatal loops that initially involve the ventral striatum may progressively transition to recruit more dorsal parts of the striatum in SRAs and other impulse control disorders (Brewer & Potenza, 2008).

Increased OFC/vmPFC as well as striatal activation in addictive disorders are in line with the general notion of an increased reward sensitivity (Balodis & Potenza, 2015; García-García et al., 2014; Luijten et al., 2017) and an overvaluation of rewarding stimuli in addiction (Schultz, 2011). Recently, the OFC has been conceptualized as providing a cognitive map of task/state space that enables inference of relationships that are not directly observed (Murray & Rudebeck, 2018; Stalnaker et al., 2015; Wilson et al., 2014; Yu et al., 2020). Such a neural representation of stimuli, actions, value, and other sensory features could explain OFC’s role in decision-making as a comparison of different values at each task state which may be over-valuated in addiction. Rewarding stimuli may include disorder-specific stimuli as well as secondary rewards (e.g., money). Importantly, this seems to be true for the anticipation of rewards, or in the case of risk-taking, the decision phase as identified by this review. In contrast, studies focusing on reward delivery suggest that individuals with SRAs exhibit a neural overvaluation of drug-related rewards and an undervaluation of nondrug rewards in response to reward reception (Gradin et al., 2014); albeit studies on neural responses to secondary rewards are somewhat inconclusive (Balodis & Potenza, 2015).

A further similarity corresponds to findings regarding the ventral ACC, although studies reporting risk-related alterations in this area are scare. Specifically, activity in the ventral ACC seems to be attenuated in both addiction types, but especially in SRAs. The ventral ACC is involved in hedonic evaluation during decision-making (Bush et al., 2000; Park et al., 2011; Phan et al., 2002; Vogt et al., 2005). Animal studies on the ventral ACC show that it is implicated in maintaining heightened arousal in reward contexts (Rudebeck et al., 2014). Reduced processing of risky stimuli in SRAs and NSRAs may represent apathetic behavior seen in individuals with addictions. A study not included in this review also reported attenuated ventral ACC activity on *occasional* stimulant users (Reske et al., 2015). Ventral ACC activity may be, therefore, already altered in individuals not showing the full-blown symptomatology of addiction. This speculation needs to be further investigated in longitudinal or family studies.

Additional risk-related alterations in activity were reported by several studies and included temporal and occipital regions as well as the cerebellum. Data on these regions is, however, scarce and mixed regarding the direction of alterations. Therefore, no concluding evidence can be obtained.

### Limitations, Implications and Future Directions

At this juncture, open questions remain and more research is needed in order to validate the preliminary findings gathered in this review. Interpretation of the results from this review are limited by several factors. The most important factors to note are (1) variability in methods (utilized tasks and neuroimaging approaches) and (2) heterogeneity in included disorders. Specifically, we compared different kind of tasks in different populations. While most SRA studies employed standard decision-making under uncertainty tasks (e.g., Balloon Analogue Risk Task, Iowa Gambling Task, Risky Gains Task; c.f. Table 2), the majority of NSRA studies used self-developed tasks and probability discounting tasks. Therefore, it is possible that the lack of evidence for altered DLPFC activity in NSRAs (and for altered IFG activity in SRAs) is related to differences in tasks used. Future studies may compare individuals with SRAs to those with NSRAs in one study in order to examine potential differences and similarities more directly. In particular, studies may use standardized tasks or a task battery and use these tasks in both addiction types, ideally in one study. Moreover, due to the sparsity of studies, we included both fMRI and PET studies. Although both methods measure cerebral blood flow, there are differences between the two methods. Most important to note is that fMRI usually has a higher resolution compared to PET imaging.

The second factor adding complexity to this review is the heterogeneity of included disorders. Specifically, we reviewed different SRA types, with different substances underlying the addiction, and with different substances expressing distinct psychopharmacological profiles. It is likely that these different SRAs may also have different neural profiles (depending on pharmacological properties of the specific substance), which we could not identify in this review.

Moreover, most reviewed studies were cross-sectional in nature. More longitudinal studies are needed in order to investigate whether alterations in risky decision-making processes are a cause or a consequence of addictive disorders. In addition, within-subject designs may also reveal predictors for relapse or recovery as shown by the study conducted by Blair et al. (2018).

It should also be noted that our conclusions are based on a small number of studies. We identify a lack of studies specifically with regard to investigating neural correlates of NSRAs. Therefore, meta-analytic techniques in order to quantitatively support our conclusions could not be applied. Future single studies as well as study syntheses may focus on brain networks related to risk-related decision-making in addiction. For example, several single fMRI studies as well as reviews on brain structure point to an aberrant frontostriatal network in SRAs (Hampton et al., 2019; Jentsch & Taylor, 1999; Suckling & Nestor, 2017). It may be of interest to investigate different networks under risky situations.

Future studies may also investigate potential differences between decisions under risk and decisions under uncertainty in these populations. In the current review, we could not identify alterations specific to either risk-taking type, which may be owed to the sparsity of studies (c.f. Table 2). Further task properties may also influence risk-related brain activation. For example, the presence of potential losses may yield different brain activation patterns compared to situations where negative trial outcomes have no consequences at all or only lead to smaller positive gains compared to positive trial outcomes. In healthy individuals, the possibility of facing losses indeed modulates brain activity (Mohr et al., 2010). It is beyond the scope of this review to investigate such potential task effects which are worth investigating in addiction in the future. An overview on identified limitations and suggestions for future directions may be found in Table 3.

**Table 3:** Limitations of the examined studies and the review itself and suggestions for future directions

**Limitations**	**Suggestions for future directions**
Differences in utilized tasks do not allow for definite conclusions about differences and similarities in neural correlates of risk-related decision-making in SRA and NSRAs	- Especially in the domain of NSRAs more studies which assess decision-making under uncertainty may be conducted - Conversely, in the domain of SRAs, more studies which assess decision-making under risk may be conducted - For direct comparison, studies may use a task battery of different risk-related decision-making tasks and use these tasks in both addiction types, ideally in one study
Conclusions of this review are limited by the fact that the sample sizes of reviewed studies was rather low and ranged from 12 to 75 participants in the group of interest. Studies on SRAs included a median of 21.5 (*M* = 28) participants, whereas studies on NSRAs included a median of 15 (*M* = 18) participants. These sample sizes are generally associated with low statistical power (Poldrack et al., 2017; Szucs & Ioannidis, 2020)	- Studies with larger sample sizes may be conducted - A priori power analysis may be conducted; for example with Neuropower (neuropowertools.org)
Evidence gained by the current review is correlational in nature. We cannot draw conclusions about the cause of aberrant risk-related neural activations	- Longitudinal and/or family studies may be conducted
We summarized findings on altered activity in specific brain regions in isolation. However, it is known that brain regions work together	- Brain network analyses may be conducted

## Conclusions

This systematic review highlights similarities and differences in neural correlates of risk-related decision-making between different addiction types (SRAs and NSRAs). Here, we gather exploratory evidence of altered risk-related processes, including hyperactivity in the OFC and the striatum in both addiction types. As characteristic for both addiction types, these brain regions may represent an underlying mechanism of suboptimal decision-making. In contrast, decreased DLPFC activity may be specific to SRAs and decreased IFG activity could only be identified for NSRAs. While findings regarding the precuneus and posterior cingulate point to elevated risk-related activity in SRAs, findings regarding these regions are mixed but rather point to attenuated activation patterns in NSRAs. Additional scarce evidence suggests decreased ventral ACC activity and increased dorsal ACC activity in both addiction types. However, these preliminary findings should be interpreted with caution due to a lack of studies is this field. Future research is needed to evaluate the findings gathered by this review.

## Supplementary Information

Below is the link to the electronic supplementary material.

Supplementary file1 (DOCX 20 KB)

Supplementary file2 (DOCX 644 KB)

## Data Availability

All of the reviewed studies are publicly available.
