# Supplementary material for: A Systematic Review on Common and Distinct Neural Correlates of Risk-taking in Substance-related and Non-substance Related Addictions
Source: Neuropsychol Rev. 2022 Jul 30;33(2):492–513. doi: 10.1007/s11065-022-09552-5 (PMC10148787; doi:10.1007/s11065-022-09552-5)
Supplement: Supplementary file 1 — Supplementary file1 (DOCX 20 KB) [file 11065_2022_9552_MOESM1_ESM.docx]

**Supplementary Material**

Neural Correlates of Risk-Taking in Addiction Disorders:

A Systematic Literature Review

**Table S1**

*Overview of Paradigms Employed by the Reviewed Studies*

| **Task** | **Description** |
| --- | --- |
| Balloon Analogue Risk Task | Participants are presented with a balloon and required to press a button to either inflate the balloon or to “cash out”. The balloon can either grow larger or explode. A larger balloon is related to an increase in earned money, but also to a greater probability of explosion. If participants choose to cash-out prior to the balloon exploding then they collect the money earned for that trail, but if the balloon explodes earnings for that trial are lost. In fMRI versions of the task, usually, control balloons are included which are not associated with reward or explosions, but serve as control for visual and motor aspects of the task. Risky decision-making on this task is operationalized by calculating the average number of pumps on unexploded balloons. In fMRI tasks, usually, the modulation of brain activity with increasing number of pumps (i.e. greater risk) of active versus control balloons is investigated. |
| Cambridge Risk Task | On each trial, participants are presented with an array of six boxes of two different colors (blue and red, for example). The participants’ task is to guess where a token is hidden by selecting one of the two colors. The odds of winning varied across trials (usually, 3:3, 4:2, 5:1). For example, the 1:5 ratio indicates that there is one blue box and 5 red boxes yielding a 16.6% chance that the blue choice would be correct, the 2:4 ratio reflected a 33.3% chance blue was correct, and the 3:3 ratio indicated that the odds were 50%.  Risky decision-making on this task is operationalized as choosing the color that is underrepresented in a current trial. |
| Cups Task | Participants are presented with several cups on each trial. The cups task includes a gain domain and a loss domain and participants are instructed to win as much money as possible in the gain domain and to lose as little money as possible in the loss domain. For both domains, participants are required to choose between a risky option and a safe option. The safe option is to win or lose $1 for sure, whereas the risky option is associated with a probability of 0.20, 0.33, or 0.50 of a larger win or loss ($2, $3, or $5).  Participants either play the loss domain or the gain domain condition where condition type is predetermined and participants know which condition they play. |
| Iowa Gambling Task | On each trial, participants are presented with four decks of cards, two of which (A and B) are associated with high gains of money, but which also lead to greater losses in the long run. Thus, choosing cards from these decks can be considered to indicate risky behavior. In contrast, the remaining two decks (C and D) are associated with smaller gains, which, in the long run, lead to greater gains. Choosing cards from these decks can be considered to indicate safe behavior. Participants begin with a specific amount of money, which is usually not real. |
| Probability Discounting Task | Participants are presented with two choice options, where one option is associated with a fixed small monetary reward and another options is associated with a larger monetary reward with a low probability of occurrence. The occurrence of the larger reward is based on a probability value presented to participants (e.g. 100 percent 10 $ versus 20 percent 15$). Participants have to indicate which option they choose. |
| Risky Gains Task | Participants are presented successively with three numbers (20, 40, and 80) in ascending order. Each number is shown for one second and if the participant presses a button when the number is presented on the screen, he or she receives that number of points shown. The goal of the task is to earn as many points as possible. Points are usually exchanged for money at the end of the task. Choosing 20 is the safe option and 40 and 80 are risky options. If a participant presses the button when 20 appears, then the trial ends and the participant receives 20 points. If the participant bypasses 20, then the 40 option appears. On some trials, the participant loses 40 points and the trial terminates. Otherwise, the participant has the option to press the button and to earn 40 points. If the 40 points option is bypassed, then 80 appears. Like the 40 option, sometimes the participant immediately loses 80 points and the trial terminates. Otherwise, the participant presses the button, receives 80 points and the trial terminates. The probabilities of punishment trials are set so that choosing the same option on each trial would produce the same final points in total. Thus, there is no advantage in choosing risky (40 or 80 points) over safe (20 points) options. |
| **Self-developed tasks:** |  |
| Brevers et al., 2015: Modified Card Deck Paradigm | On each trial, participants are presented with a deck of cards containing red and blue cards. The paradigm has two conditions: a (1) risky condition, where the number of red and blue cards contained in the deck are written on the deck, such that participants are aware of the distribution of red and blue cards and a (2) ambiguous condition, where the distribution of red and blue cars are not known. For each condition, three options were given: to bet on either the red or the blue card, or to choose a sure payoff that was however much lower than the potential payoff from a bet choice. |
| Dong & Potenza, 2015: Risky decision-making and gambling task | This task includes a risk-taking stage as well as a risky decision-making stage. Participants are presented with two rows of cards with four cards each either depicted in red or yellow color. Each card has a number written on it. Red cards indicate the number on it may be won, while yellow cards indicate the number written on it may be lost. The probability and magnitude of the gains/losses are manipulated to create advantageous/disadvantageous risky selections. Advantageous risk-taking means the sum of numbers on red cards (win) are larger than those on yellow cards (loss). In the risk-taking stage, the participant’s task is to choose whether to play with row one or row two. In the risky decision-making stage, the participant’s task was to indicate which of the four cards is red (i.e., a winning card). |
| Gilman et al., 2015: Lane Risk-Taking Task | Participants are presented with two squares. One of the squares has a question mark written beneath it. If the participant chooses the square without the question mark (the ‘safe’ square), they are guaranteed to win $0.25. If the participant chooses the square with the question mark (the ‘risky’ square), they could win $1.00 or $5.00, but they could also risk to losing $1.00 or $5.00. Fifty percent of risky squares result in wins and 50 percent result in losses, but participants are not aware of these probabilities. |
| Miedl et al., 2010, 2014: Experimental blackjack task | This paradigm is based on the card game blackajack. Participants are presented with regular playing cards. In low-risk trials, participants either start with 12 or 13 points against 7,8,9, or 10 points of the dealer, whereas in high-risk trials participants start with 15 or 16 points against sed on ectivelyregualtion 7,8,9, or 10 points of the dealer. The probability of losing while drawing a card was 0.34 and 0.56 over low-risk and high-risk trials, respectively. In all trials a hit is more advantageous than a stand. A bet is 5€ or 1€. |
