# Supplementary figures and images for: A Systematic Review on Common and Distinct Neural Correlates of Risk-taking in Substance-related and Non-substance Related Addictions
Source: Neuropsychol Rev. 2022 Jul 30;33(2):492–513. doi: 10.1007/s11065-022-09552-5 (PMC10148787; doi:10.1007/s11065-022-09552-5)

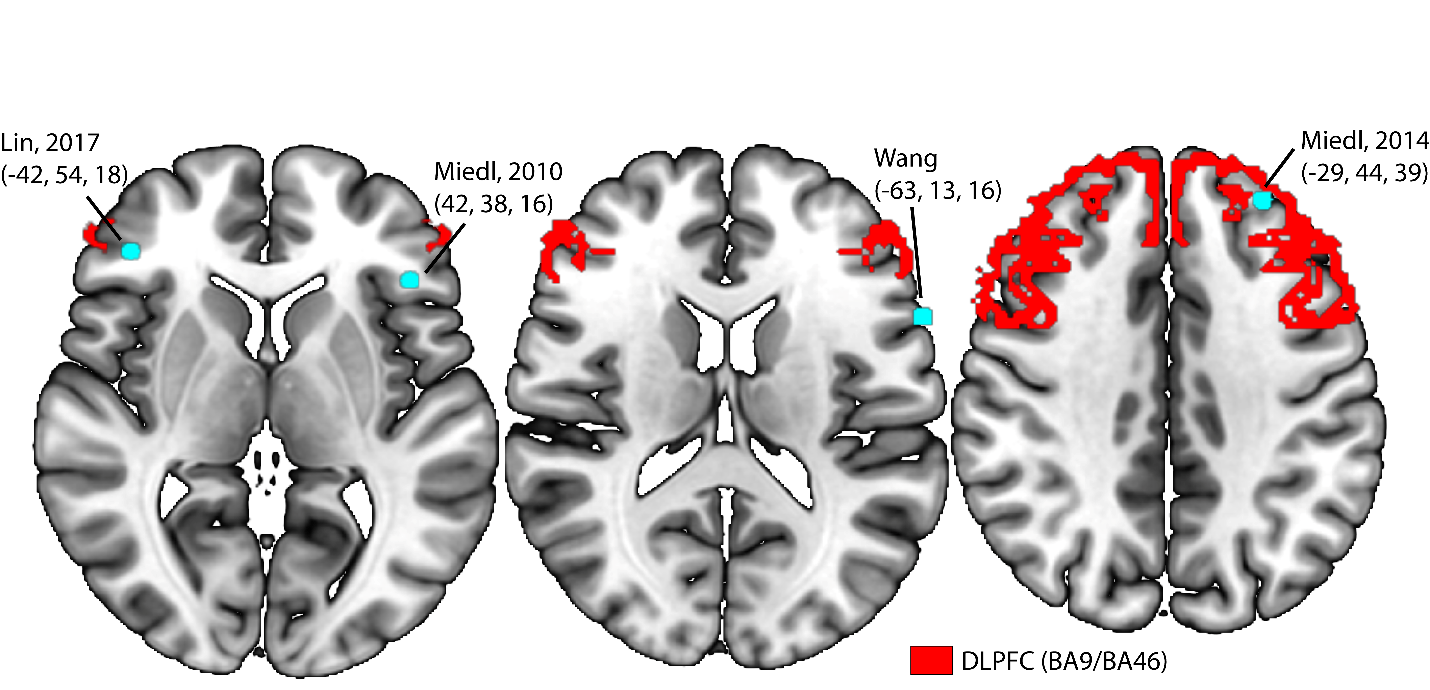

Supplement: Supplementary file 2 — Supplementary file2 (DOCX 644 KB) [file 11065_2022_9552_MOESM2_ESM.docx]
